# ^68^Ga-DOTATATE PET/CT imaging of indeterminate pulmonary nodules and lung cancer

**DOI:** 10.1371/journal.pone.0171301

**Published:** 2017-02-09

**Authors:** Ronald Walker, Stephen Deppen, Gary Smith, Chanjuan Shi, Jonathan Lehman, Jeff Clanton, Brandon Moore, Rena Burns, Eric L. Grogan, Pierre P. Massion

**Affiliations:** 1 Medical Imaging Service, Tennessee Valley VA Healthcare System, Nashville, Tennessee, United States of America; 2 Department of Radiology and Radiological Sciences, Vanderbilt University Medical Center, Nashville, Tennessee, United States of America; 3 Vanderbilt-Ingram Cancer Center, Nashville, Tennessee, United States of America; 4 Department of Surgery, Vanderbilt University Medical Center, Nashville, Tennessee, United States of America; 5 Department of Pathology, Microbiology and Immunology, Vanderbilt University Medical Center, Nashville, Tennessee, United States of America; 6 Department of Medicine, Division of Hematology/Oncology, Vanderbilt University Medical Center, Nashville, Tennessee, United States of America; 7 Department of Surgery, Tennessee Valley Healthcare System, Nashville, Tennessee, United States of America; 8 Department of Medicine, Division of Pulmonary, Allergy and Critical Care Medicine, Vanderbilt University Medical Center, Nashville, Tennessee, United States of America; 9 Pulmonary Critical Care Section, Medical Service, Tennessee Valley Healthcare System, Nashville, Tennessee, United States of America; Kyungpook National University School of Medicine, REPUBLIC OF KOREA

## Abstract

**Purpose:**

^18^F-FDG PET/CT is widely used to evaluate indeterminate pulmonary nodules (**IPNs**). False positive results occur, especially from active granulomatous nodules. A PET-based imaging agent with superior specificity to ^18^F-FDG for IPNs, is badly needed, especially in areas of endemic granulomatous nodules. Somatostatin receptors (SSTR) are expressed in many malignant cells including small cell and non-small cell lung cancers (**NSCLCs**). ^68^Ga-DOTATATE, a positron emitter labeled somatostatin analog, combined with PET/CT imaging, may improve the diagnosis of IPNs over ^18^F-FDG by reducing false positives. Our study purpose was to test this hypothesis in our region with high endemic granulomatous IPNs.

**Methods:**

We prospectively performed ^68^Ga-DOTATATE PET/CT and ^18^F-FDG PET/CT scans in the same 30 patients with newly diagnosed, treatment-naïve lung cancer (*N* = 14) or IPNs (*N* = 15) and one metastatic nodule. ^68^Ga-DOTATATE SUVmax levels at or above 1.5 were considered likely malignant. We analyzed the scan results, correlating with ultimate diagnosis via biopsy or 2-year chest CT follow-up. We also correlated ^68^Ga-DOTATATE uptake with immunohistochemical (**IHC**) staining for SSTR subtype 2A (**SSTR2A**) in pathological specimens.

**Results:**

We analyzed 31 lesions in 30 individuals, with 14 (45%) being non-neuroendocrine lung cancers and 1 (3%) being metastatic disease. McNemar’s result comparing the two radiopharmaceuticals (p = 0.65) indicates that their accuracy of diagnosis in this indication are equivalent. ^68^Ga-DOTATATE was more specific (94% compared to 81%) and less sensitive 73% compared to 93%) than ^18^F-FDG. ^68^Ga-DOTATATE uptake correlated with SSTR2A expression in tumor stroma determined by immunohistochemical (**IHC**) staining in 5 of 9 (55%) NSCLCs.

**Conclusion:**

^68^Ga-DOTATATE and ^18^F-FDG PET/CT had equivalent accuracy in the diagnosis of non-neuroendocrine lung cancer and ^68^Ga-DOTATATE was more specific than ^18^F-FDG for the diagnosis of IPNs. IHC staining for SSTR2A receptor expression correlated with tumor stroma but not tumor cells.

## Introduction

There were an estimated 221,000 newly diagnosed cases of lung cancer in the US in 2014, with an estimated 158,000 deaths. Lung cancer is the leading cause of cancer death in the US and worldwide. The five-year relative cancer survival of all stages and all types of lung cancer combined (2002–2008) is 17%.[1] While ^18^F-fluorodeoxyglucose (^**18**^**F-FDG**) PET/CT is approved and widely used for staging of lung cancer, and for initial diagnosis of an indeterminate pulmonary nodule (**IPN**), the accuracy of ^18^F-FDG PET/CT for diagnosis of IPNs depends on many factors, including regional exposures and infectious lung diseases. Regionality can result in variable test accuracy by influencing false positive (**FP**) and false negative (**FN**) results.[2, 3] Lesion size below 1 cm and low proliferative lung cancers can lead to FN results from low metabolic uptake.[4, 5] FP results occur from infectious or inflammatory foci, most infamously granulomatous nodules. Granulomatous nodules occur in much of the United States (**US**), especially the Ohio/Mississippi River Valley and the Southwestern US. These benign nodules arise from a variety of soil fungi, such as *Histoplasma capsulatum*, endemic in our region. We have reported [6] that ^18^F-FDG PET/CT is very sensitive (92% true positive, **TP**) for the diagnosis of IPNs in our region, but with a specificity (true negative, **TN**) of only 40%, mostly due to FPs from granulomatous nodules, with similar results from others.[2, 7] Given our 40% regionally low specificity of ^18^F-FDG PET/CT for IPNs, many clinicians have chosen serial CT exams to follow IPNs with Fleischner guidelines.[8] Using either Fleischner criteria for a prevalent nodule, or Lung-RADS criteria for a screening-detected nodule, a solid IPN must show no growth for 2 years by CT to be considered benign.[9, 10]

During this follow-up period, malignant nodules could theoretically metastasize, resulting in a missed chance for a cure. An option to interval CT follow-up is to perform a tissue diagnosis at discovery, preferred for high risk patients with solid nodules 0.8 cm or greater in diameter. Below this size, the likelihood of a successful, definitive biopsy diminishes with the decreasing size of the IPN.[11] Clearly there is a great need for a PET-based imaging agent to improve the 40% specificity of ^18^F-FDG in areas of endemic granulomatous lung nodules without sacrificing ^18^F-FDG’s approximately 90% sensitivity for lung cancer.

Somatostatin receptors (**SSTR**) are a family of G protein-coupled receptors whose signaling alter hormonal secretion, modulate apoptosis, and regulate cellular proliferation.[12–14] These receptors are expressed in many normal and malignant cells, including both small cell (**SCLC**) and non-small cell lung cancers (**NSCLC**). SSTRs signal canonically through cAMP, cGMP, and phosphotyrosine phosphatases leading to downstream AKT and ERK1/2 signaling changes, increasing apoptosis and decreasing cell proliferation in multiple cell types. Radiolabeled SSTR2 analogs are utilized for imaging tumors, especially neuroendocrine tumors.[15] SSTRs, have little expression in normal lung tissue, but have increased expression in lung cancer correlating with SSTR2A IHC staining [16, 17] and ^111^In-Pentetreotide uptake.[18] We tested the hypothesis that PET/CT imaging with a somatostatin analog labeled with ^68^Ga, a positron emitter, might demonstrate superior accuracy compared to ^18^F-FDG for diagnosis of IPNs in our region by improving specificity with similar sensitivity. To test our hypothesis, we compared the ^18^F-FDG and ^68^Ga-labeled 1,4,7,10-tetraazacyclododecane-N,N9,N99,N999-tetraacetic acid (^**68**^**Ga-DOTATATE**) PET/CT scans in patients with newly detected, untreated IPNs or lung cancer at VA Tennessee Valley Healthcare System (**VATVHS**) in Nashville, TN.

## Methods

This investigation was performed with VATVHS IRB approval (#974), and with oversight of the VATVHS Radioactive Drug Research Committee. All subjects gave their own written informed consent. Consents were recorded in the subjects’ electronic healthcare record. All consents were obtained with IRB review and approval. ^68^Ga-DOTATATE imaging was for correlation of tumor or nodule uptake with final diagnosis via tissue diagnosis or 2-year follow-up in all 30 subjects, but not for altering standard of care management. Our measured dosimetry of ^68^Ga-DOTATATE is previously reported, with similar results reported by others.[19, 20]

### Patient selection

Inclusion criteria required adults aged ≥30 years able to provide informed consent, with a newly diagnosed IPN (noncalcified pulmonary nodule 7–20 mm diameter) or a newly diagnosed, untreated lung cancer, and able to complete the dual imaging protocol. Both genders were eligible, though the study reflects the predominately male gender of the VA population, 28 males and one female.

### Imaging protocol

Radiopharmaceutical preparation was described previously. [19] All ^18^F-FDG PET/CT scans were standard-of-care. The patients fasted for at least 8 hours, limiting physical exertion for 24 hours, before ^18^F-FDG PET/CT scanning. Insulin-dependent diabetics withheld insulin for ≥8 hours before ^18^F-FDG IV injection of 600 MBq, range 360–890 MBq (16.4 mCi, range 9.7–24.0 mCi), with the activity adjusted for body weight. ^18^F-FDG imaging started 60 min (range 55–104) after injection, with fasting blood sugar averaging 119 mg/dL (range 71–267 mg/dL). Each subject received 185 MBq, range 166–222 MBq (5 mCi, range 4.5–6.0 mCi), of ^68^Ga-DOTATATE intravenously, imaged 60 min (range 45–118 min) after injection. Excluding subjects for dosimetry measurements, [19] the subjects emptied their bladders immediately before and after scanning, and were encouraged to hydrate and urinate frequently for 12 hours. All imaging was performed with an integrated emission/transmission scanner (Discovery VCT, GE Healthcare, Waukesha, WI, USA) from vertex to mid-thigh with a low-dose CT without contrast used for anatomic localization and attenuation correction, followed by emission imaging over the same regions with four minute bed positions, 3D mode, using a 128 matrix, as previously reported.[19] Imaging interval between ^18^F-FDG and ^68^Ga-DOTATATE scans averaged 11 days, range 3–29, with no intervening treatment.

### Immunohistochemistry

Immunohistochemical (IHC) staining for somatostatin receptor 2A (SSTR2A, Biotrend, Schwabhausen, Germany) was performed on 5 μM formalin-fixed paraffin-embedded sections as described [21] and interpreted visually. Of 22 lesions with tissue confirmation (14 NSCLCs, 8 benign lesions), 12 had IHC staining with scoring by intensity of membranous or cytoplasmic staining (no stain = 0, weak staining = 1, moderate staining = 2, strong staining = 3) and extent of stained cells (<5% = 0, 5–25% = 1, 26–50% = 2, and 51–75% = 3, >75% = 4).[22, 23] SSTR2A immunoreactivity was evaluated in all cellular elements.

### Molecular imaging data analysis

Imaging results were analyzed with the Xeleris 2^™^ workstation (GE Healthcare, Waukesha, WI, USA). Standard 1 mL regions of interest were measured to obtain the maximum standardized uptake values (**SUVmax**) normalized to lean body mass for primary tumors. All subjects in our study were at high risk for lung cancer as defined by the National Lung Screening Trial, [24] and either had known lung cancer or indeterminate but high risk nodules based on CT appearance. Our analysis solely focuses on comparing uptake of our two radiopharmaceuticals for discriminating benign from malignant disease, not on using the often invaluable contribution of the CT portion of the PET/CT, for the purposes of this pilot study.[25] Accordingly, a cut-off of SUVmax of ^18^F-FDG of ≥ 2.5 was arbitrarily used for diagnosis of cancer.[6] An SUVmax of ≥ 1.5 was retrospectively and empirically chosen for ^68^Ga-DOTATATE to optimize overall accuracy for our population’s lung cancer prevalence, which was *a-priori* assumed to be approximately 50%. Final diagnosis was determined by either pathological tissue diagnosis or 2-year radiographic surveillance by CT. CT follow-up was diagnostic of a benign solid nodule if the nodule was stable or decreasing in size at 24 months, had developed interim benign calcifications, or had resolved.

Contingency tables using the above criteria were created independently for ^18^F-FDG-PET and ^68^Ga-DOTATATE-PET accuracy to diagnose cancer in all patients. Sensitivity, specificity, positive and negative predictive values, with 95% confidence intervals, with McNemar’s chi-square. All analyses were performed in Stata v12 (College Station, TX.)

## Results

There were no observed serious adverse events from ^68^Ga-DOTATATE. Long term follow-up of lab values from routine medical care, when available, revealed no evidence of delayed toxicity.

### Patient characteristics

Of 31 lesions, 15 were malignant. Average age was 61.2 years). Median lesion size was 22 mm (inter-quartile range, 15–39 mm). Twelve of 31 lesions were larger than 30 mm. Fourteen of 15 (93%) had NSCLC, and 1 had metastatic transitional cell carcinoma. The other 15 patients had 16 benign nodules. Two subjects had 2 distinctly separate IPNs, each analyzed independently with regards to diagnostic test results.

In one subject with a resected IPN, a benign 1.5 cm granuloma, an incidental microscopic focus of bronchus-associated lymphoid tissue (BALT) lymphoma was found in the surgical specimen, separate from the granulomatous nodule. We have included the imaging findings of this granulomatous nodule, negative for significant uptake by both ^18^F-FDG and ^68^Ga-DOTATATE, in the analysis of other benign nodules. Therefore, from 30 patients, we report the findings of 14 primary lung cancers and 16 benign nodules, and one patient with a metastatic nodule from transitional cell carcinoma. We obtained primary tumor tissue in 12 patients to correlate immunohistochemistry with imaging.

### Molecular imaging characteristics of ^68^Ga-DOTATATE PET

Prevalence of malignancy among the 31 lesions was 48%, higher than our reported prevalence for IPNs, due to the inclusion of high risk subjects with lesion diameter > 2 cm in this pilot trial.

**Fig 1:**
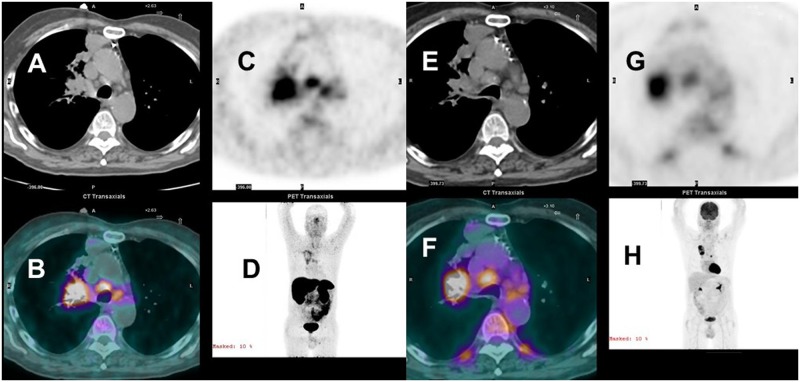
^68^Ga-DOTATATE (A-D) and ^18^FDG PET/CT (E-H) concordant staging stage IIIB squamous cell carcinoma. Concordant uptake in the right upper lobe tumor, right hilum and mediastinal adenopathy. Axial CT images (A&E) at mid chest with emission (C&G) and fused images (B&F). Anterior 3D maximum intensity images, D&H.

**Fig 2:**
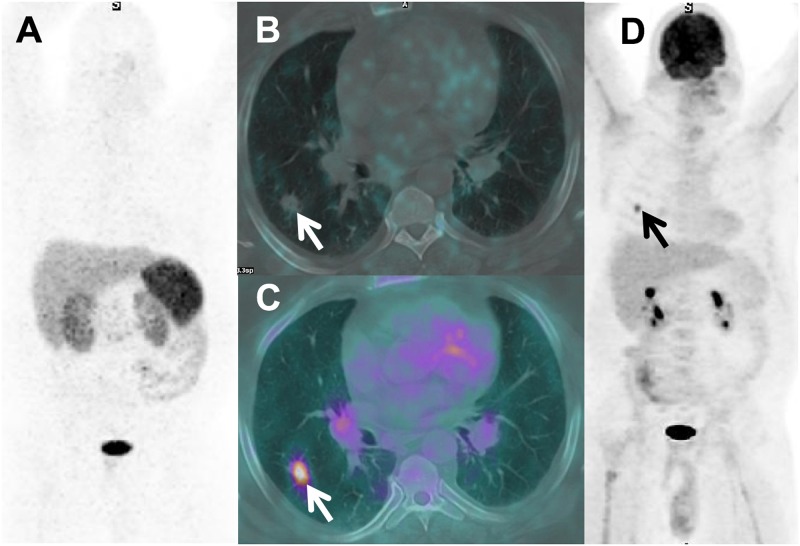
Discordant image, FP ^18^F-FDG, TN ^68^Ga-DOTATATE PET/CT. Axial CT (A) with a spiculated IPN. Fused ^18^F-FDG PET/CT (B) with IPN intense uptake (SUVmax 5.8) vs. ^68^Ga-DOTATATE (C) with no visible uptake (SUVmax 0.90). Comparison ratios of the SUVmax of the nodule to normal lung and aortic blood pool for 18F-FDG were 11.8 and 6.4, respectively, with corresponding values of ^68^Ga-DOTATATE being 2.2 and 1.5. Biopsy revealed inflammatory cells; nodule resolved on CT follow-up.

^68^Ga-DOTATATE was more specific (94% compared to 81% for ^18^F-FDG) and less sensitive (73% compared to 93%) than ^18^F-FDG (Table 1). When comparing the ability of each test to differentiate benign disease and malignancy, both tests characterized the same 24 lesions correctly and both characterized two lesions incorrectly (Table 2). Of two lesions incorrectly classified, one was a false negative by both tests and was a partially differentiated adenocarcinoma and one benign lesion was a false positive by both modalities. Three malignant lesions, two adenocarcinomas and one metastatic transitional cell carcinoma, were classified correctly by ^18^F-FDG and incorrectly by ^68^Ga-DOTATATE. Two lesions, both benign, were classified incorrectly by ^18^F-FDG and correctly by ^68^Ga-DOTATATE. There was no significant difference between the two imaging modalities in their overall diagnostic accuracy by McNemar’s test (p = 0.65). Detailed imaging characteristics of ^18^F-FDG and ^68^Ga-DOTATATE imaging are summarized in Table 3. Overall uptake in normal lung and mediastinal tissues was low and similar between ^68^Ga-DOTATATE and ^18^F-FDG (Fig 1). Two inflammatory benign nodules had intense ^18^F-FDG uptake (Fig 2), but were not avid with ^68^Ga-DOTATATE. The area under the receiver operating characteristic curve (**AUC**) for ^18^F-FDG was 0.87 (95%CI: 0.76 to 0.99), and for ^68^Ga-DOTATATE was 0.84 (95%CI: 0.70 to 0.97), SUVmax cut-point, not significantly different (chi-square; p = 0.58). The results of the ^68^Ga-DOTATATE and ^18^F-FDG PET/CT for diagnosing and staging of IPNs and lung cancer in this investigation were statistically equivalent.

**Table 1:** Performance comparison of ^18^F-FDG and ^68^Ga-DOTATATE.

N = 31	^18^F-FDG (95% CI)	^68^Ga-DOTATATE (95% CI)
SENS (%)	93.3 (68.1–99.8)	73.3 (44.9–92.2)
SPEC (%)	81.3 (54.4–96.0)	93.8 (69.8–99.8)
PPV (%)	82.4 (56.6–96.2)	91.7 (61.5–99.8)
NPV (%)	92.9 (66.1–99.8)	78.9 (54.4–93.9)
Accuracy (%)	87.3 (0.76–0.99)	83.5 (0.70–0.97)

^18^F-FDG SUVmax ≥ 2.5 or ^68^Ga-DOTATATE SUVmax ≥ 1.5 defines a positive study.

SENS, sensitivity; SPEC, specificity; PPV, positive predictive value; NPV, negative predictive value; SUVmax, maximum standardized uptake value; CI, confidence intervals

**Table 2:** Contingency table categorizing the diagnostic accuracy of ^18^F-FDG and ^68^Ga-DOTATATE PET/CT with McNemar’s chi-square test.

	^18^F-FDG Correct	^18^F-FDG Incorrect
^**68**^**Ga-DOTATATE Correct**	24	2
^**68**^**Ga-DOTATATE Incorrect**	3	2

McNemar’s chi-square (1df) = 0.20; p = 0.65S

**Table 3:** Summary of imaging characteristics (30 patients, 31 lesions).

Subj ID	Lesion diameter (cm)	^18^F-FDG SUVmax	^68^Ga-DOTATATESUVmax	Age (y)	Fasting blood sugar mg/dL	Diagnosis
2	1.6	1.0	0.4	60	267	Benign
3	1.5	1.3	0.9	46	124	Benign
25	1.5	4.5	0.6	79	104	Metastatic
40	2.3	5.0	2.5	78	136	SCC
68	7.0	6.1	1.9	60	88	Benign
70 (IPN1)	2.4	0.8	1.2	46	103	Benign
70 (IPN2)	1.2	1.9	1.0	46	103	Benign
76	1.0	2.2	1.4	72	89	Benign
85	6.0	13.6	3.8	72	112	SCC
93	2.0	1.3	0.8	68	109	Benign
95	4.1	3.5	1.2	57	97	Benign
100	2.1	13.9	3.2	65	71	SCC
101	3.1	3.7	3.4	59	93	SCC
109	3.9	1.2	0.8	40	97	Benign
120 (IPN1)	2.0	3.4	0.8	53	125	Benign
120 (IPN2)	3.0	0.86	1.2	53	125	Benign
129	1.4	0.8	1.2	64	104	Benign
137	5.5	6.5	1.6	65	130	ADC
138	2.2	5.2	0.8	50	112	ADC
146	6.9	15.0	1.5	63	101	ADC
153	3.8	5.1	2.1	73	102	ADC
162	3.2	5.6	1.8	67	241	SCC
164	1.5	9.7	2.6	64	110	ADC
179	1.1	0.7	1.0	63	116	Benign
182	1.0	1.4	0.8	46	104	Benign
183	1.0	0.5	0.5	49	92	Benign
186	1.8	5.1	1.3	70	98	ADC
191	7.0	11.2	2.1	62	88	SCC
192	4.4	2.3	1.0	62	108	ADC
204	3.1	15.3	2.5	65	141	SCC
218	1.5	0.7	0.8	83	107	Benign

Note: See Tables 1 and 2, and the Discussion, for analysis of these results.

### Localization and correlation of SSTR2 immunostaining pattern in tumors and benign lesions with *in vivo* ^68^Ga-DOTATATE PET imaging

**Fig 3:**
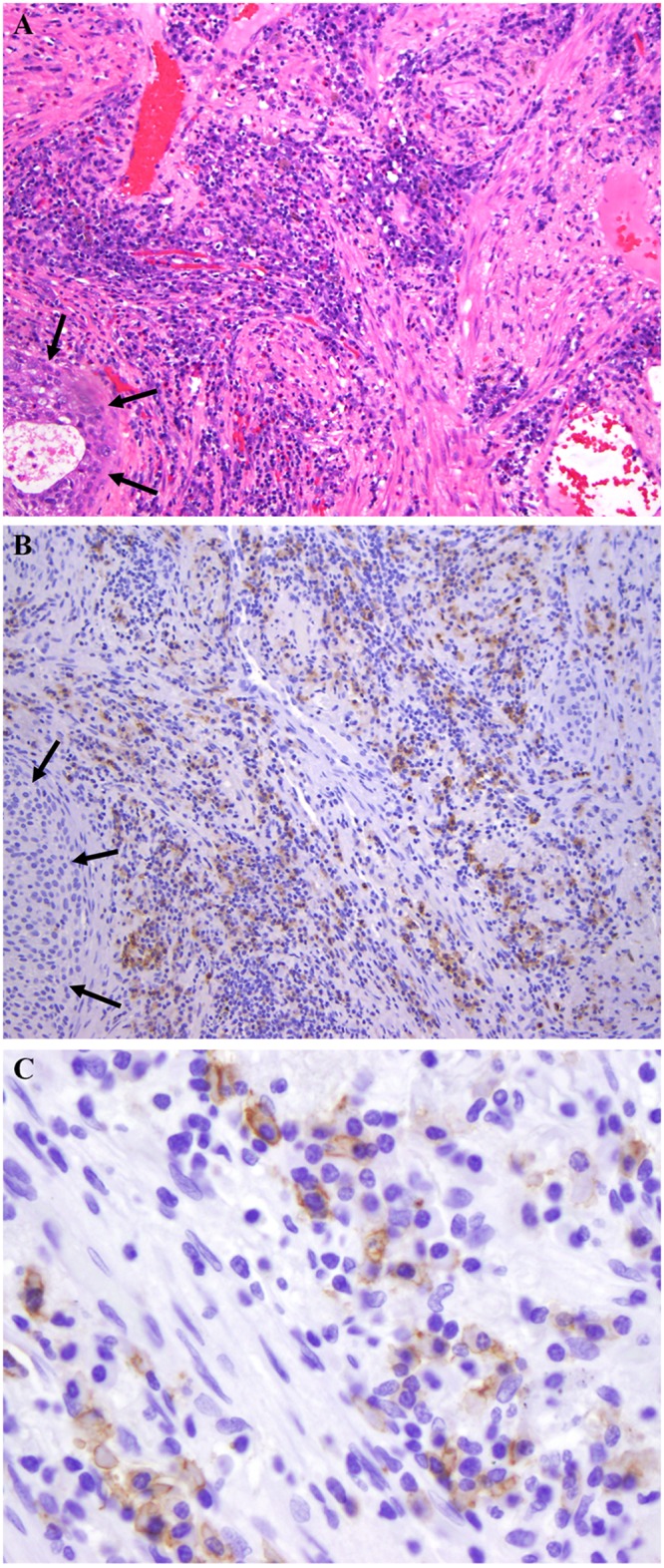
Inflammatory cells within non-neuroendocrine tumor. A. Hematoxylin and eosin stain of squamous cell carcinoma (black arrows) with abundant plasma cells. B. SSTR2A IHC stain showing negative tumor staining (black arrows) but positive staining in inflammatory cells (original magnification 100X). C. High power field shows tumor (black arrows) with SSTR2A IHC membranous staining in plasma cells (white arrows).

To further define SSTR2A receptor expression in lung cancer, we examined SSTR2A expression by IHC staining in our malignant and benign nodules (Table 4). Although we had no neuroendocrine lung cancers, or lung cancers with neuroendocrine differentiation, in our 30 patients, some degree of neuroendocrine differentiation is reported in one-third of non-small cell lung cancers.[26] Thus, the ^68^Ga-DOTATATE uptake in our patients was not due to uptake in either classic neuroendocrine lung cancers or lung cancers with neuroendocrine differentiation. Cancer diagnosis was confirmed by tissue in all cases. Qualitative analysis of SSTR2A IHC staining revealed no staining in tumor cells, with staining limited to support stroma and/or inflammatory cells (Fig 3), including histiocytes, plasma cells, lymphocytes, dendritic cells, and neutrophils, or in pericytes and endothelial cells of small blood vessels.

**Table 4:** SSTR2 IHC staining results—12 biopsies.

Patient ID	Diagnosis	Biopsy Source	Staining Intensity	Percent Cells Staining	IHC Score	Cells with IHC Staining	SUVmax ^68^Ga-DOTATATE	SUVmax ^18^F-FDG
153	ADC	Resection	2	<5%	0	Inflam	2.1	5.1
137	ADC	Resection	0	0	0	None	1.6	6.5
138	ADC	Resection	1	<5%	0	NeoV	0.8	5.2
204	SCC	Biopsy	1	30%	2	Inflam, NeoV	2.5	15.3
120	Benign	Biopsy	0	0	0	None	0.8	3.4
70	Benign	Biopsy	0	0	0	None	1.2	0.8
129	Benign	Biopsy	0	0	0	None	1.2	0.8
101	SCC/ADC	Resection	2	20%	2	Inflam	3.4	3.7
192	ADC	Resection	1	<5%	0	Inflam	1.0	2.3
40	SCC	FNA Bx	0	0	0	none	2.5	5.0
164	ADC	FNA Bx	0	0	0	none	2.6	9.7
100	SCC	FNA Bx	0	0	0	none	3.2	13.9

ADC, adenocarcinoma; SCC, squamous cell cancer; SCC/ADC, squamous cell cancer with adenosquamous features; Poorly diff, poorly differentiated lung cancer; Resection, tissue via surgical resection; Biopsy, core or wedge biopsy; FNA Bx, fine-needle aspiration biopsy; Inflam, inflammatory infiltration; LF, lymphoid follicles; NeoV, neovascular endothelium, pericytes.

Of the 9 NSCLC specimens, 5 demonstrated SSTR2A IHC positivity, with 3 of 4 with negative staining obtained via fine-needle aspiration (FNA) samples with little tumor stroma, possibly limiting the accuracy of IHC staining. Of the 3 benign lesions, none demonstrated IHC positivity. Adequacy of IHC staining technique, including those without tumor immunoreactivity, was verified by positive immunoreactivity in an associated inflammatory cell, such as a lymphocyte or plasma cell, and by the use of positive and negative controls. To further confirm the observed findings, we tested SSTR2A IHC in tissue microarrays of 3 NSCLC cell types and in a granuloma tissue array, noting consistent results with SSTR2A immunoreactivity confined to tumor stromal or endothelial cells (S1 Fig), similar to our clinical specimens.

## Discussion

This study uniquely reports the results of a prospective trial comparing ^18^F-FDG with ^68^Ga-DOTATATE PET/CT in patients with newly diagnosed lung cancer or IPNs in patients at high risk for lung cancer. Importantly, we performed our investigation in a region with high endemic granulomatous nodules, a confounder of ^18^F-FDG PET imaging.[27] Our investigation demonstrates that ^68^Ga-DOTATATE is equivalent in accuracy to ^18^F-FDG PET/CT for diagnosis of a malignant IPN, and, in most cases, for diagnosis and staging of NSCLC (Table 1). Since ^68^Ga is a generator-based product, the equivalent accuracy of ^68^Ga-DOTATATE vs. ^18^F-FDG PET/CT suggests that, upon validation in a larger cohort, ^68^Ga-DOTATATE PET/CT could be an alternative to ^18^F-FDG PET/CT in areas where ^18^F-FDG is not available, which would include isolated areas of the US (Rocky Mountain states such as Idaho, Montana, or Wyoming) or developing countries, where access to a medical cyclotron is still limited.

Investigators have tested a variety of imaging probes to differentiate benign malignant lung nodules, including other somatostatin receptor imaging agents, such as ^99m^Tc-depreotide. In an early report of SSTR imaging in lung cancer, Kwekkeboom, et al [28] reported successful imaging of small cell lung cancer using ^123^I-Tyr-3-octreotide in 5 of 8 subjects (62.5%).

Kahn and colleagues [29] compared ^18^F-FDG PET with ^99m^Tc-depreotide SPECT imaging in 157 patients for staging of NSCLC with equivalent sensitivities (96% for ^18^F-FDG PET, 94% for ^99m^Tc-depreotide SPECT), though ^18^F-FDG PET specificity (71%) was superior to ^99m^Tc-depreotide SPECT (51%). Blum et al [30] reported a Phase III multi-center trial using ^99m^Tc-depreotide SPECT imaging for solitary IPNs or masses up to 6 cm in diameter; in 114 patients, ^99m^Tc-depreotide SPECT had an accuracy of 91% for discriminating benign from malignant lesions.

Halley et al [31] prospectively and consecutively enrolled 28 subjects with no prior history of cancer, comparing^18^F-FDG PET with ^99m^Tc-depreotide SPECT imaging for diagnosis of IPNs 0.8–3 cm in diameter. Combining the two tests, when either one was positive, they achieved 18/18 (100%) sensitivity for malignant diagnosis, though not significantly superior to ^18^F-FDG PET alone. Likewise, there was no significant difference in the specificity of ^18^F-FDG PET (70%) compared to ^99m^Tc-depreotide SPECT (80%).

In a prospective series, [32] Ferran, et al, compared ^18^F-FDG PET/CT and ^99m^Tc-depreotide SPECT/CT in 29 patients with IPNs. The overall accuracies were 85% for ^99m^Tc-depreotide and 96% for ^18^F-FDG PET, though not statistically different in this study. Naalsund, et al, [33] compared ^99m^Tc-depreotide SPECT or SPECT/CT imaging with histopathology in 118 subjects in a prospective multi-center trial, with 29 also receiving ^18^F-FDG PET imaging. In the subset of 29 that were compared to ^18^F-FDG PET, the results were identical between ^18^F-FDG PET and ^99m^Tc-depreotide imaging with sensitivity, specificity and accuracy of 90%, 67% and 83%.

A somatostatin analog imaging agent, ^68^Ga-1,4,7,10-tetraazacyclododecane-N,N’,N”,N”‘-tetraacetic acid-D-Phe^1^-Tyr^3^-octreotide (^68^Ga-DOTATOC), was compared with ^18^F-FDG PET in 9 NSCLC patients.[34] The mean primary tumor SUVmax for ^68^Ga-DOTATOC was 2.0 vs. 5.7 for ^18^F-FDG.

In our study, ^68^Ga-DOTATATE PET/CT and ^18^F-FDG PET/CT were statistically equivalent for diagnosing lung cancer, though there were isolated cases with discrepant findings (Fig 2). The overall accuracy of each radiopharmaceutical was 87.5%, each misclassifying 4 patients (^68^Ga-DOTATATE PET/CT misclassified the one metastatic nodule).

IHC staining positivity in our tissue specimens was not in tumor cells, but in the stroma or inflammatory cells (Table 4).[35] SSTR2A and SSTR2B can be highly expressed on a variety of other, benign cells as well, including activated macrophages, fibroblasts and the endothelium.[36–38] This phenomenon has been reported before by Reubi and colleagues [39], and several others, in a variety of tumors, where IHC staining for SSTR2A receptors was observed in the stroma, especially the neovascularity, of a variety of malignancies, and not limited to the malignant cells. Additional causes of uptake include hemangiomas, with large areas of endothelium, and active granulomatous diseases (such as acute fungal or mycobacterial infection) or a subacute to chronic bacterial pneumonia. Still, SUVmax values averaged higher for ^68^Ga-DOTATATE in lung cancer than in benign lesions (2.1 vs.0.9, respectively), as did SUVmax valued for ^18^FDG (8.1 vs. 1.7, respectively). Similarly, Herlin, et al, [40] found uptake of ^99m^Tc-depreotide in NSCLC did not correlate with IHC expression levels of SSTR2A in tumor cells, consistent with our tissue samples and microarrays (S1 Fig).

Limitations of our investigation include our small number of subjects with tissue sampling (12 of 30, 40%). The unexpected lack of granulomatous nodules may be due to study size and incomplete tissue sampling. In other series from areas of endemic histoplasmosis, granuloma prevalence was 50%–65% [3, 27, 41], and may be represented in those ^18^F-FDG and ^68^Ga-DOTATATE negative nodules that were followed by CT for 2 or more years to establish benignity. Another limitation was our predominately male population.

In conclusion, we prospectively imaged 30 patients with 31 total lesions with ^18^F-FDG and ^68^Ga-DOTATATE PET/CT (S1 Table). ^18^F-FDG and ^68^Ga-DOTATATE imaging had equivalent accuracy (87.5%), each misclassifying 4 lesions. The overall accuracy of diagnosis of NSCLC using ^68^Ga-DOTATATE and ^18^F-FDG PET/CT was equivalent (87.5%). IHC staining for SSTR2A was non-reactive in all tumor cells (none were neuroendocrine, and none demonstrated neuroendocrine differentiation on histological examination) with ^68^Ga-DOTATATE uptake likely within the tumor stroma, though averaging more in malignant lesions, suggesting a tumor/microenvironment interaction deserving further investigation. The sensitivity of ^18^F-FDG for malignancy (92.9%) was very close to our previously reported regional results for indeterminate lung nodules, [6] though the specificity (83.3%) was much higher, probably because we enrolled subjects with a known diagnosis of lung cancer as well as indeterminate lung nodules. Thus, our hypothesis that ^68^Ga-DOTATATE PET/CT imaging in our area of high endemic granulomatous nodules would be more accurate for the diagnosis of a malignant lesion than ^18^F-FDG PET/CT was not supported by our data. While ^68^Ga-DOTATATE PET/CT was more specific for the diagnosis of lung cancer than ^18^F-FDG PET/CT, it was also less sensitive. Additionally, our data demonstrate that the majority of SSTR2A receptor expression was related to tumor stroma in our non-neuroendocrine lung cancers, suggesting a tumor/stroma interaction that deserves further investigation.

## Supporting information

S1 Fig Pathological specimens and tissue microarrays with hematoxylin and eosin (H&E) and SSTR2A immunohistochemical (IHC) staining. Black arrows (when present) indicate tumor cells. Red arrows (when present) indicate IHC staining for SSTR2A in stroma and/or inflammatory cells.
(PDF)

S1 Table Complete dataset of prospective imaging of 30 patients with 31 total lesions with 18F-FDG and 68Ga-DOTATATE PET/CT. (PDF)
