# Supplementary material for: 68Ga-DOTATATE PET/CT imaging of indeterminate pulmonary nodules and lung cancer
Source: PLoS One. 2017 Feb 9;12(2):e0171301. doi: 10.1371/journal.pone.0171301 (PMC5300187; doi:10.1371/journal.pone.0171301)
Supplement: S1 Fig — Black arrows (when present) indicate tumor cells. Red arrows (when present) indicate IHC staining for SSTR2A in stroma and/or inflammatory cells. (PDF) [file pone.0171301.s001.pdf]

^{68}Ga-DOTATATE PET/CT Imaging of Indeterminate Pulmonary Nodules and Lung Cancer

Ronald Walker,[1,2,3] Stephen Deppen,[4] Gary Smith,[1,2] Chanjuan Shi,[5] Jonathan Lehman,[6] Jeff Clanton,[2] Brandon Moore,[1] Rena Burns,[3] Eric L Grogan,[4,7] Pierre P Massion[3,8,9*]

Affiliations:
[1]Medical Imaging Service, Tennessee Valley VA Healthcare System, Nashville, TN, United States of America
[2]Department of Radiology and Radiological Sciences, Vanderbilt University Medical Center, Nashville, TN, United States of America
[3]Vanderbilt-Ingram Cancer Center, Nashville, TN, United States of America
[4]Department of Surgery, Vanderbilt University Medical Center, Nashville, TN, United States of America
[5]Department of Pathology, Microbiology and Immunology, Vanderbilt University Medical Center, Nashville, TN, United States of America
[6]Department of Medicine, Division of Hematology/Oncology, Vanderbilt University Medical Center, Nashville, TN, United States of America
[7]Department of Surgery, Tennessee Valley Healthcare System, Nashville, TN, United States of America
[8]Department of Medicine, Division of Pulmonary, Allergy and Critical Care Medicine, Vanderbilt University Medical Center, Nashville, TN, United States of America
[9]Pulmonary Critical Care Section, Medical Service, Tennessee Valley Healthcare System, Nashville, TN, United States of America

Running title: ^{68}Ga-DOTATATE - Lung Nodules & Cancer

*Corresponding author
Email: pierre.massion@vanderbilt.edu

Key words: ^{68}Ga-DOTATATE; lung cancer; lung nodule; somatostatin receptor

Word count: 5842

Supporting Information 1
Pathological specimens and tissue microarrays with hematoxylin and eosin (H&E) and SSTR2A immunohistochemical (IHC) staining:

Black arrows (when present) indicate tumor cells. Red arrows (when present) indicate IHC staining for SSTR2A in stroma and/or inflammatory cells

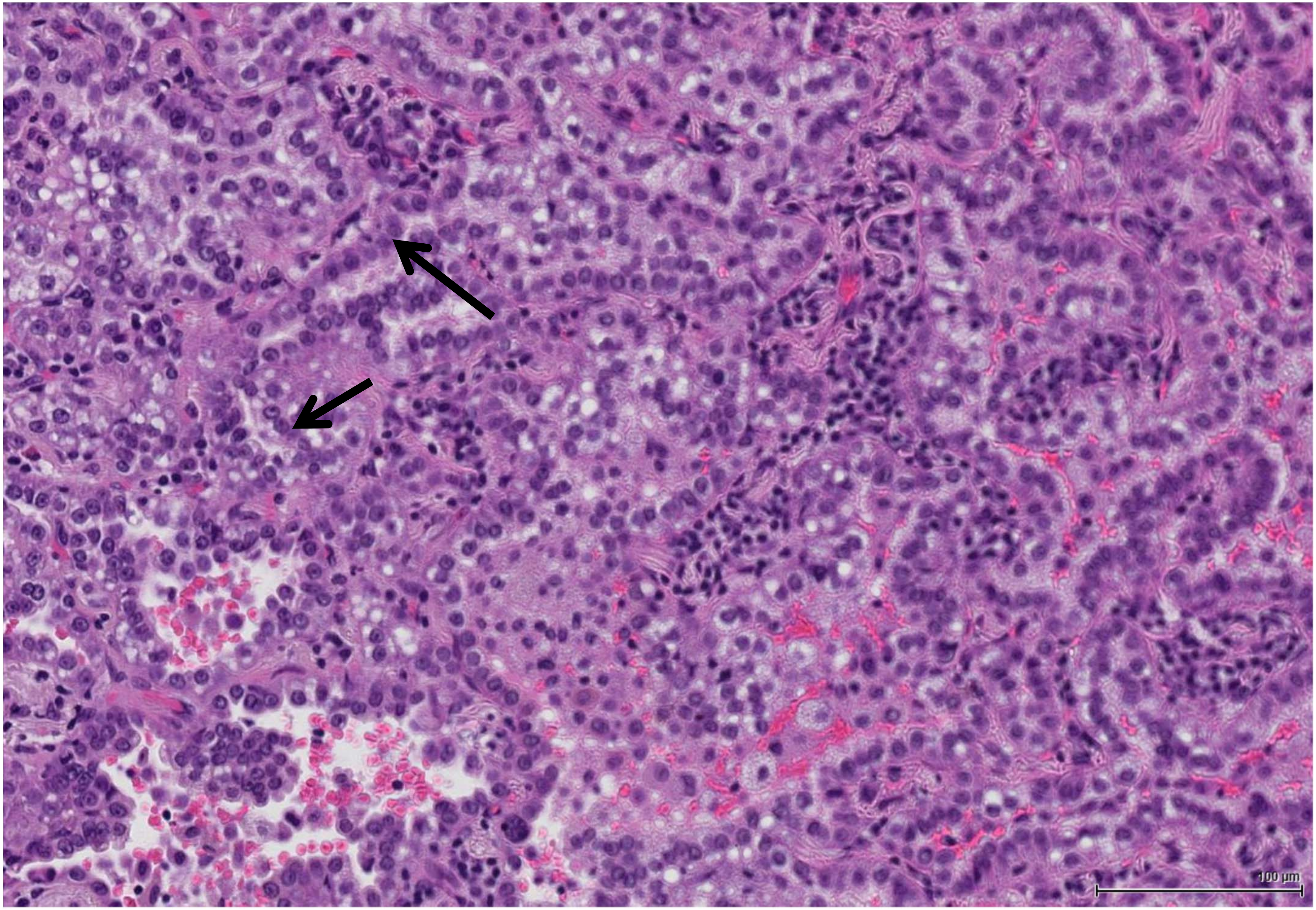

Well differentiated adenocarcinoma of the lung, tumor cells (black arrows), H&E stain.

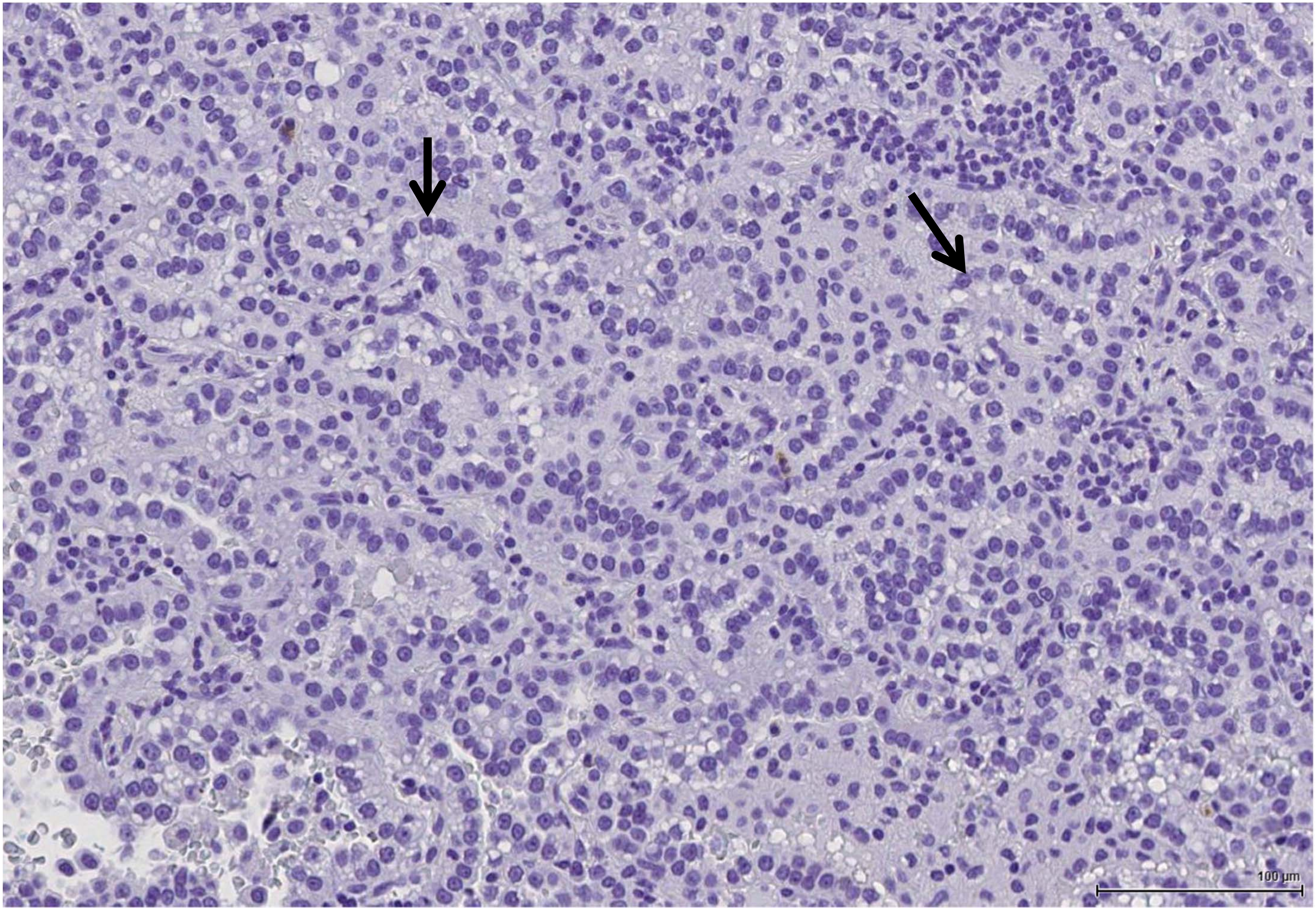

Well differentiated adenocarcinoma of the lung, tumor cells (black arrows) with no SSTR2A IHC staining.

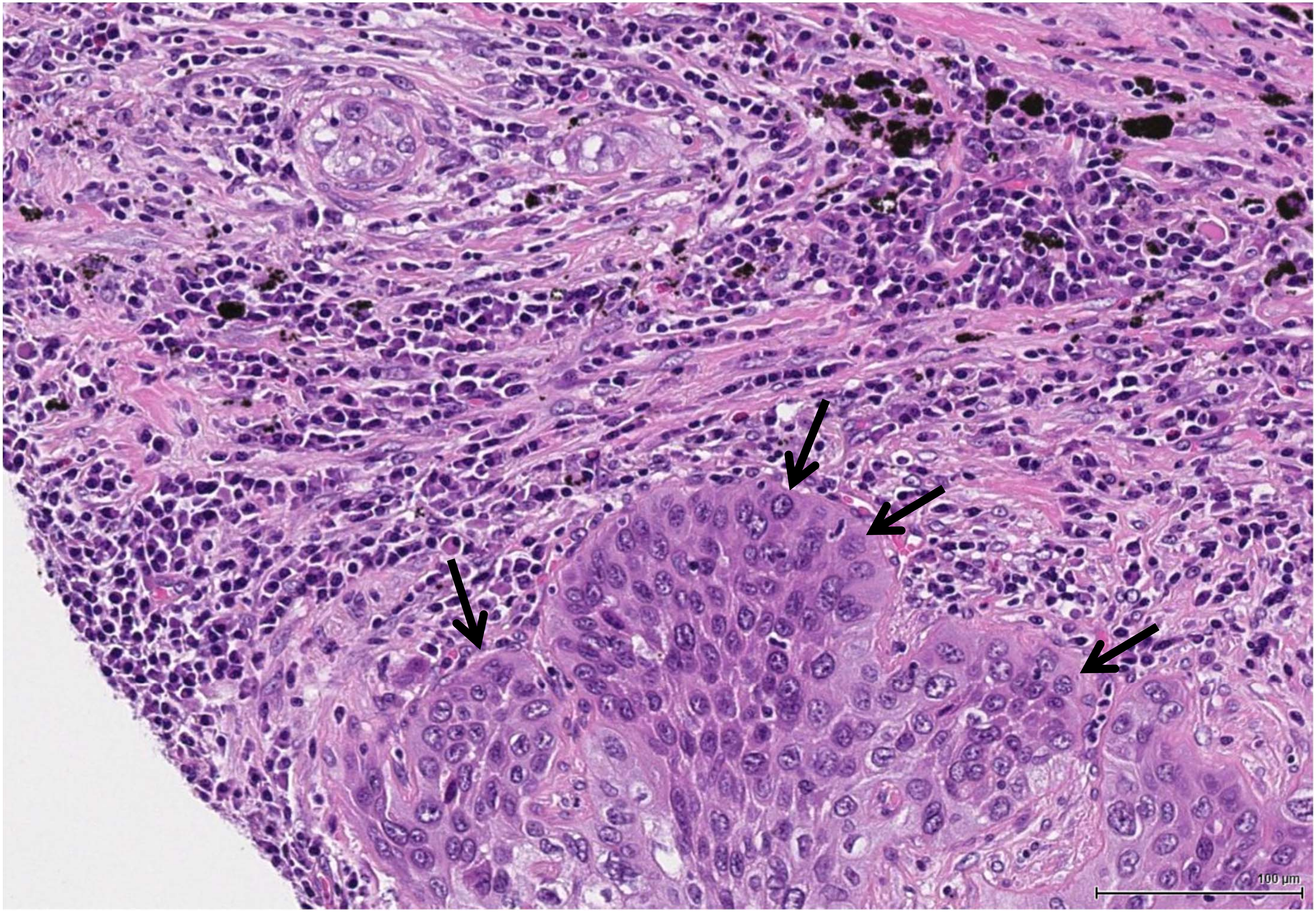

Squamous cell carcinoma of the lung. Tumor cells shown (black arrows). H&E stain.

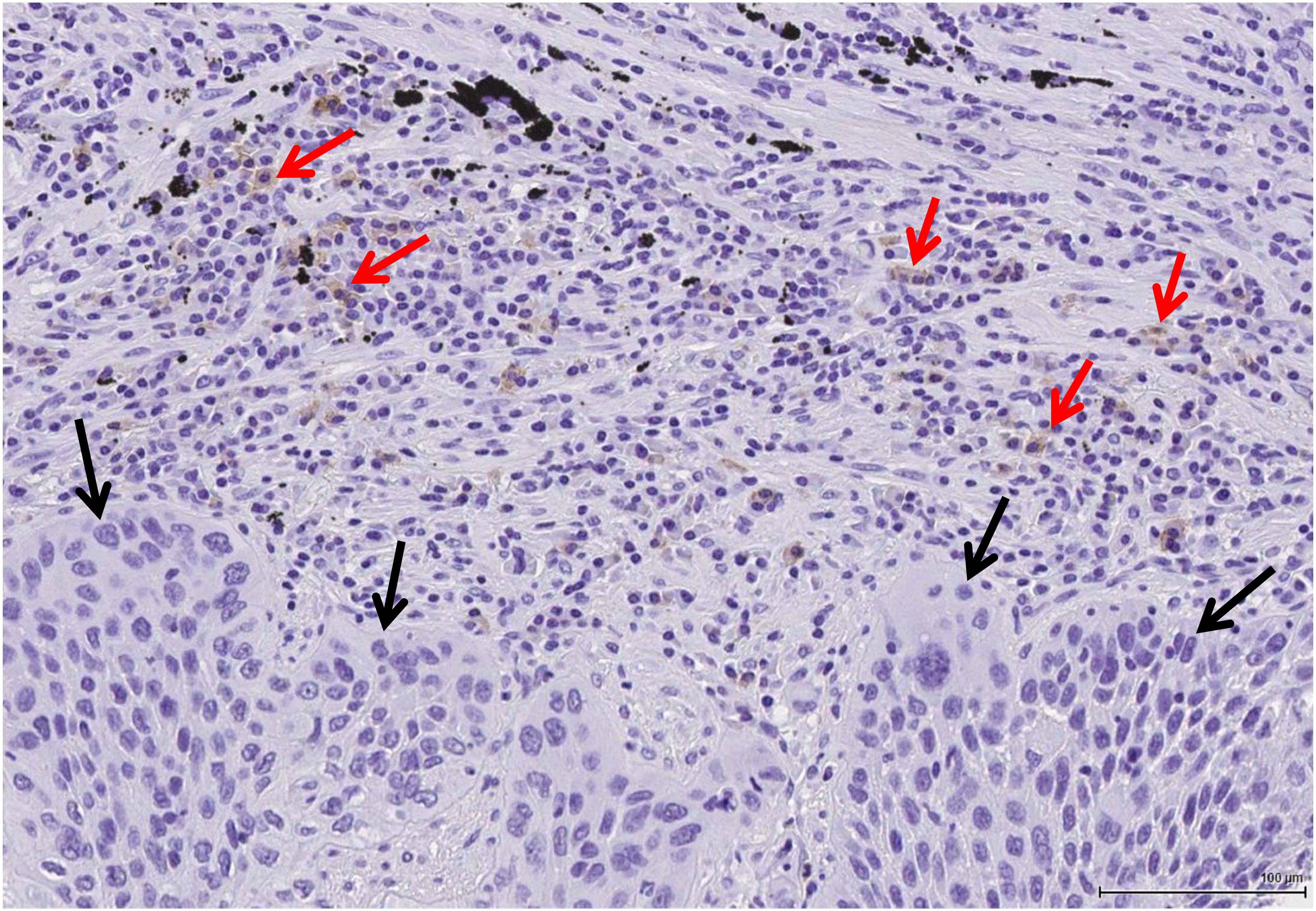

Squamous cell carcinoma of the lung. Tumor cells (black arrows) negative for SSTR2A IHC stain. Inflammatory cells (some indicated, red arrows) demonstrate IHC positivity.

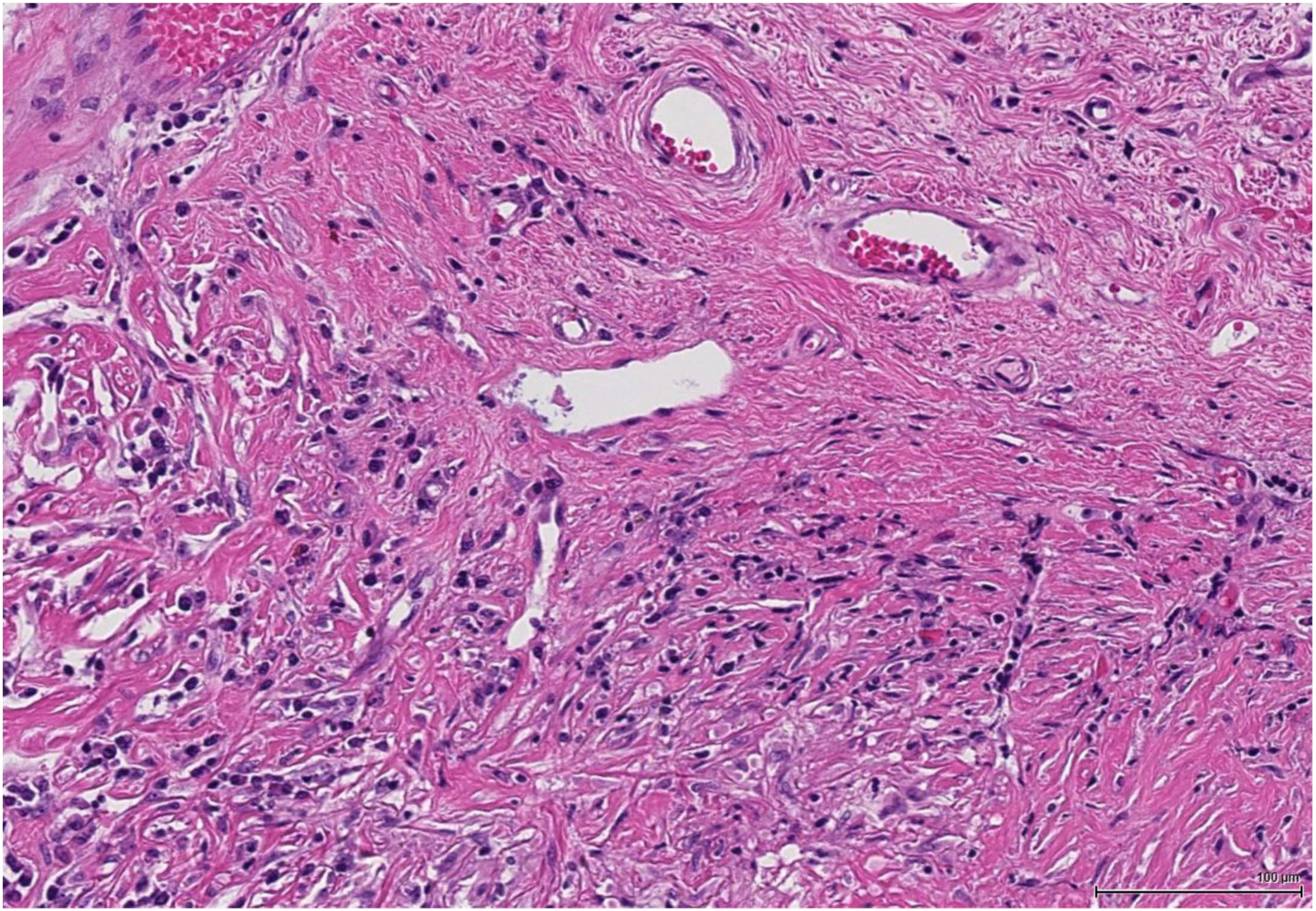

Benign granuloma. H&E stain.

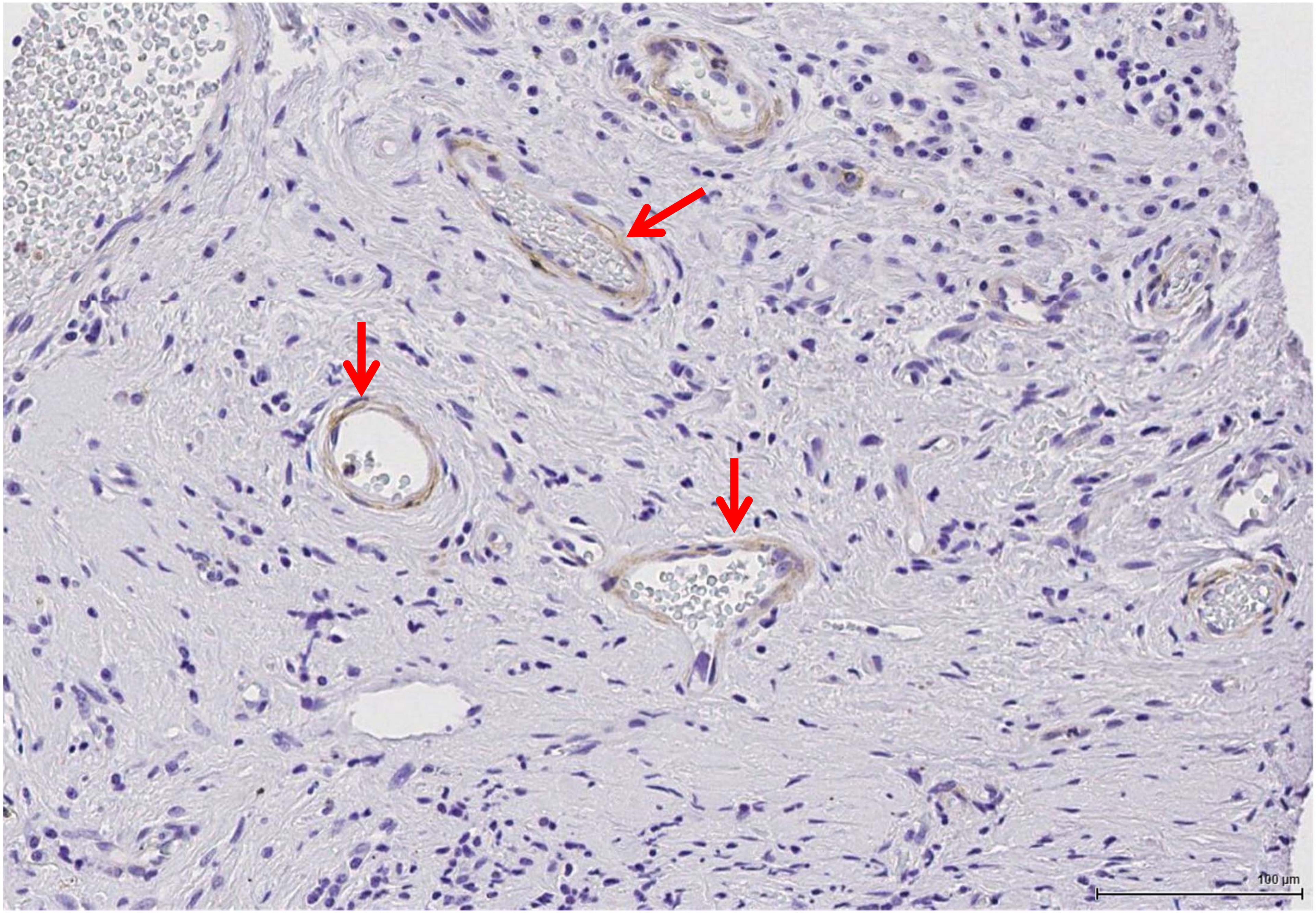

Benign granuloma with SSTR2A positivity in endothelium of small blood vessels (some indicated with red arrows)

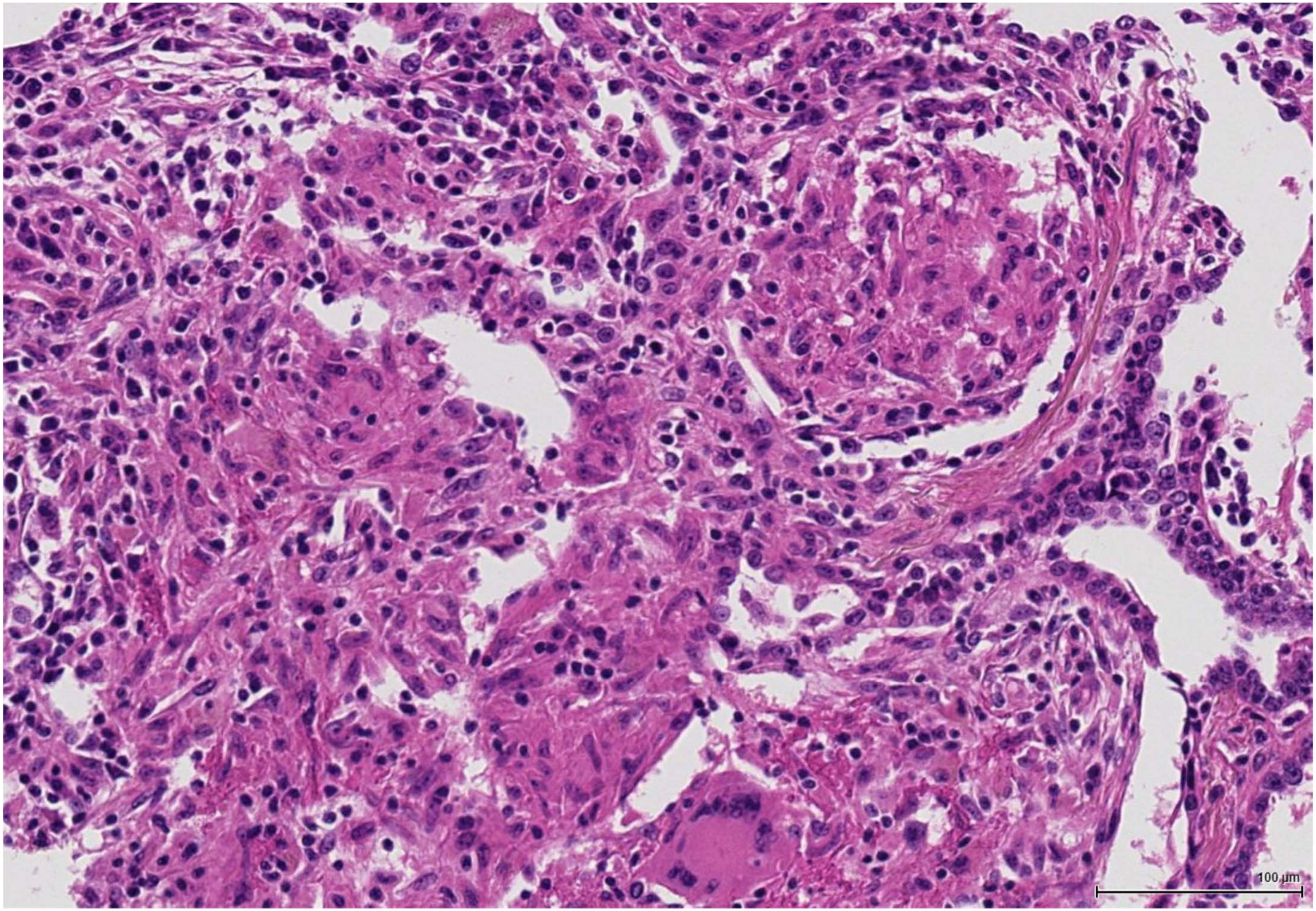

Benign granuloma. H&E stain.

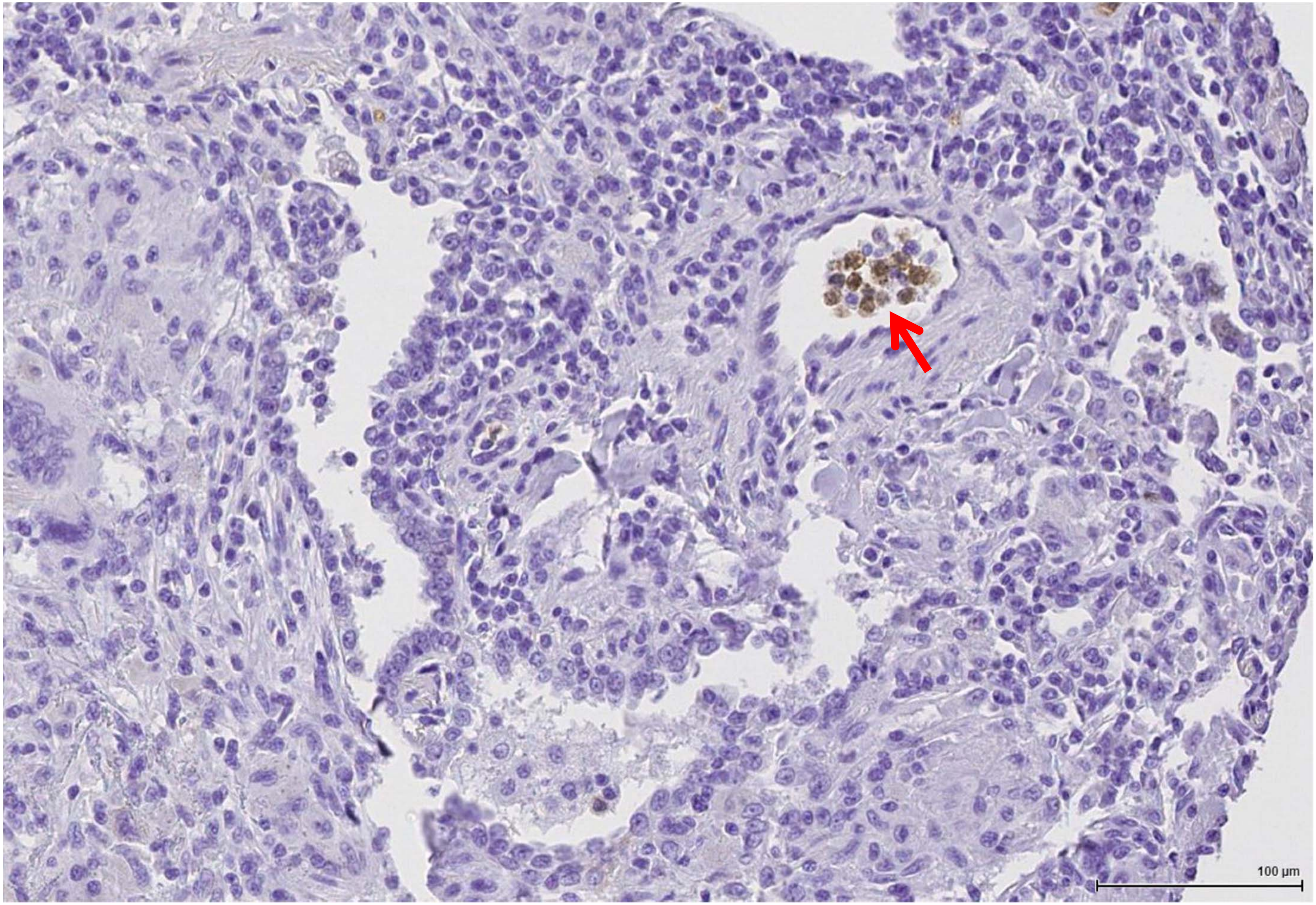

Benign granuloma with SSTR2A positivity in a cluster of inflammatory cells (red arrow).

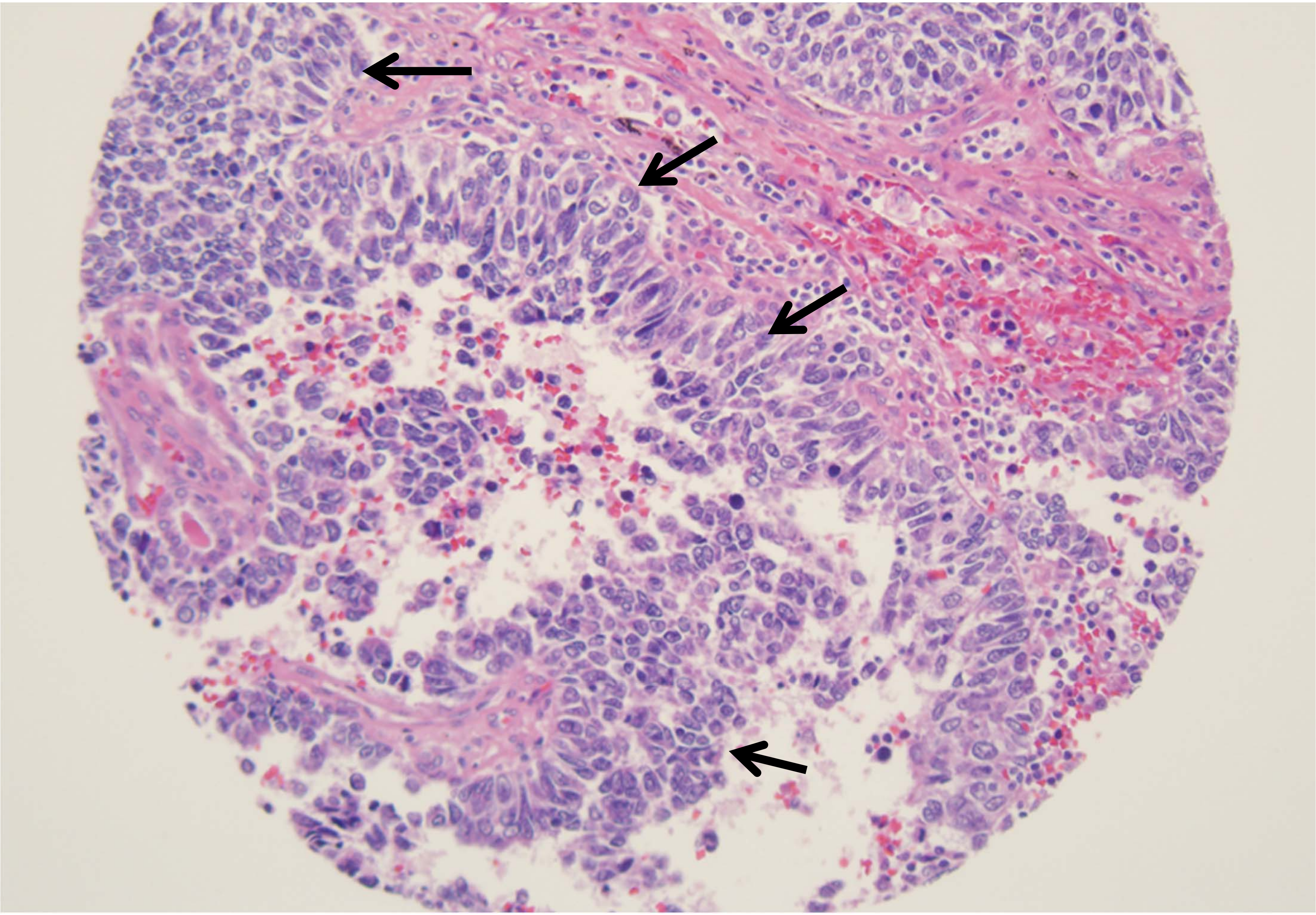

Well differentiated neuroendocrine tumor of the lung. H&E stain. Some tumor cells indicated by black arrows). Tissue microarray, not from our patients.

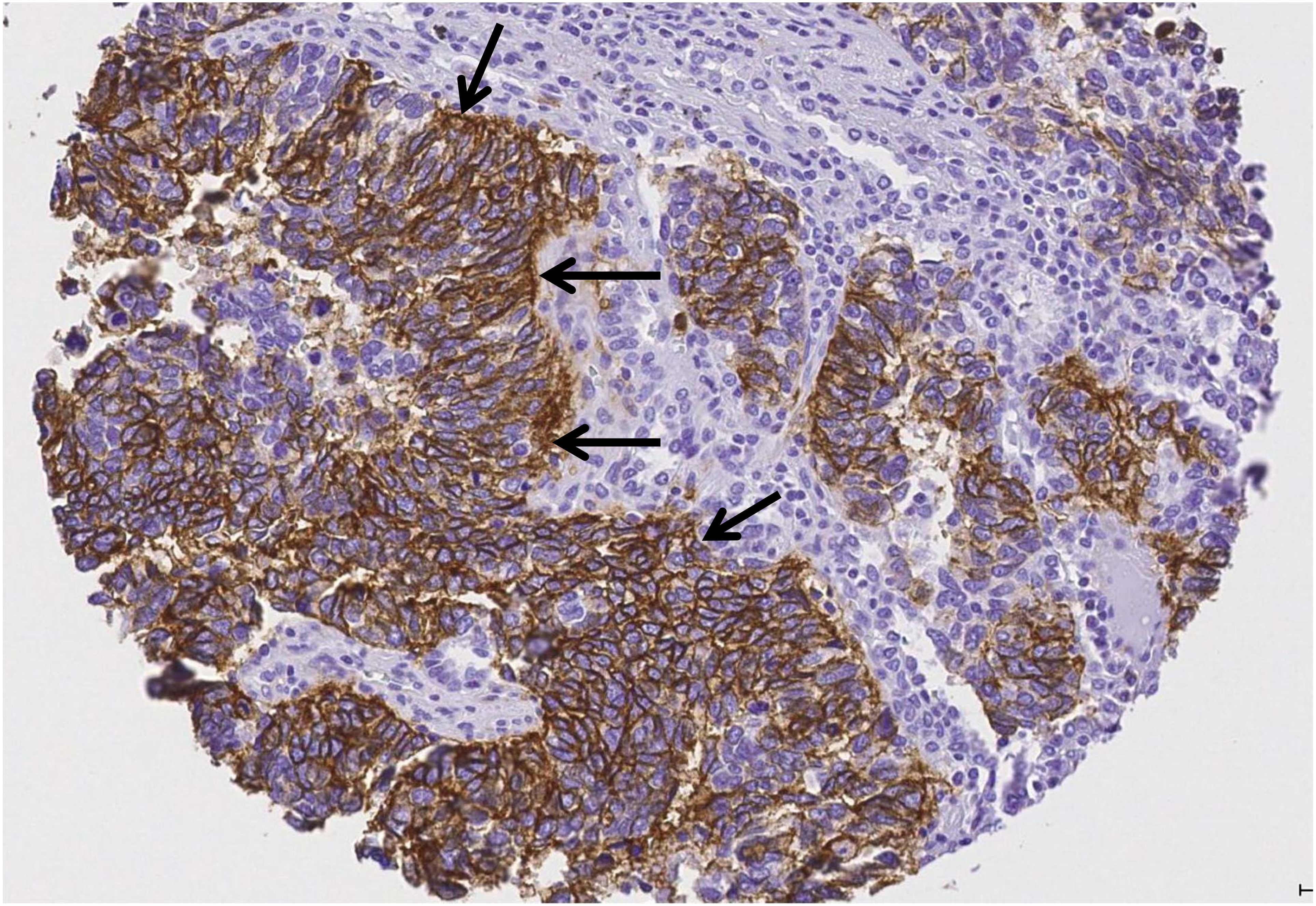

Well differentiated neuroendocrine tumor of the lung. H&E stain. Some tumor cells indicated by black arrows). Tissue microarray, not from our patients.

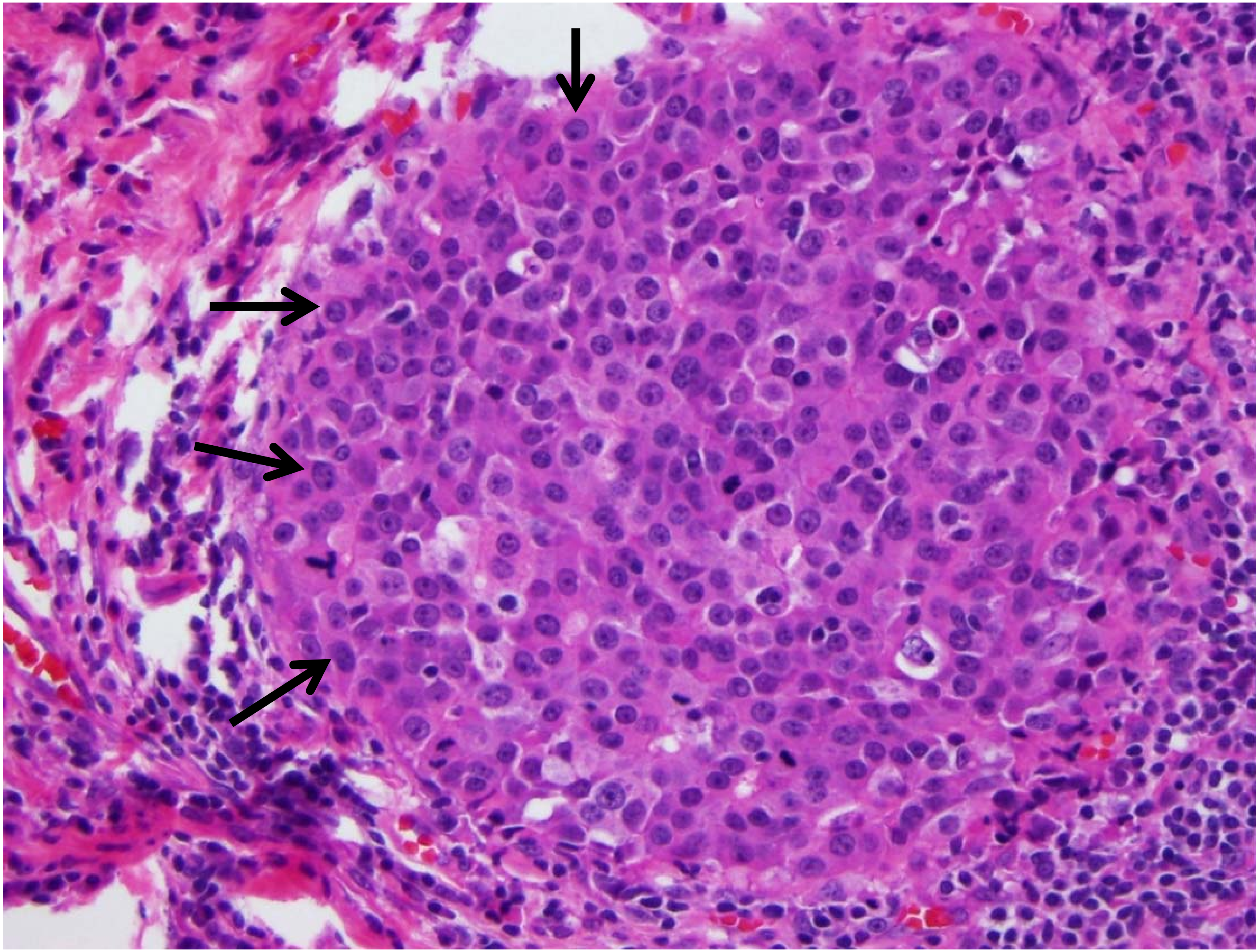

Lung adenocarcinoma with neuroendocrine differentiation. H&E stain. Some tumor cells indicated with black arrows. This example is from our patients but not this report.

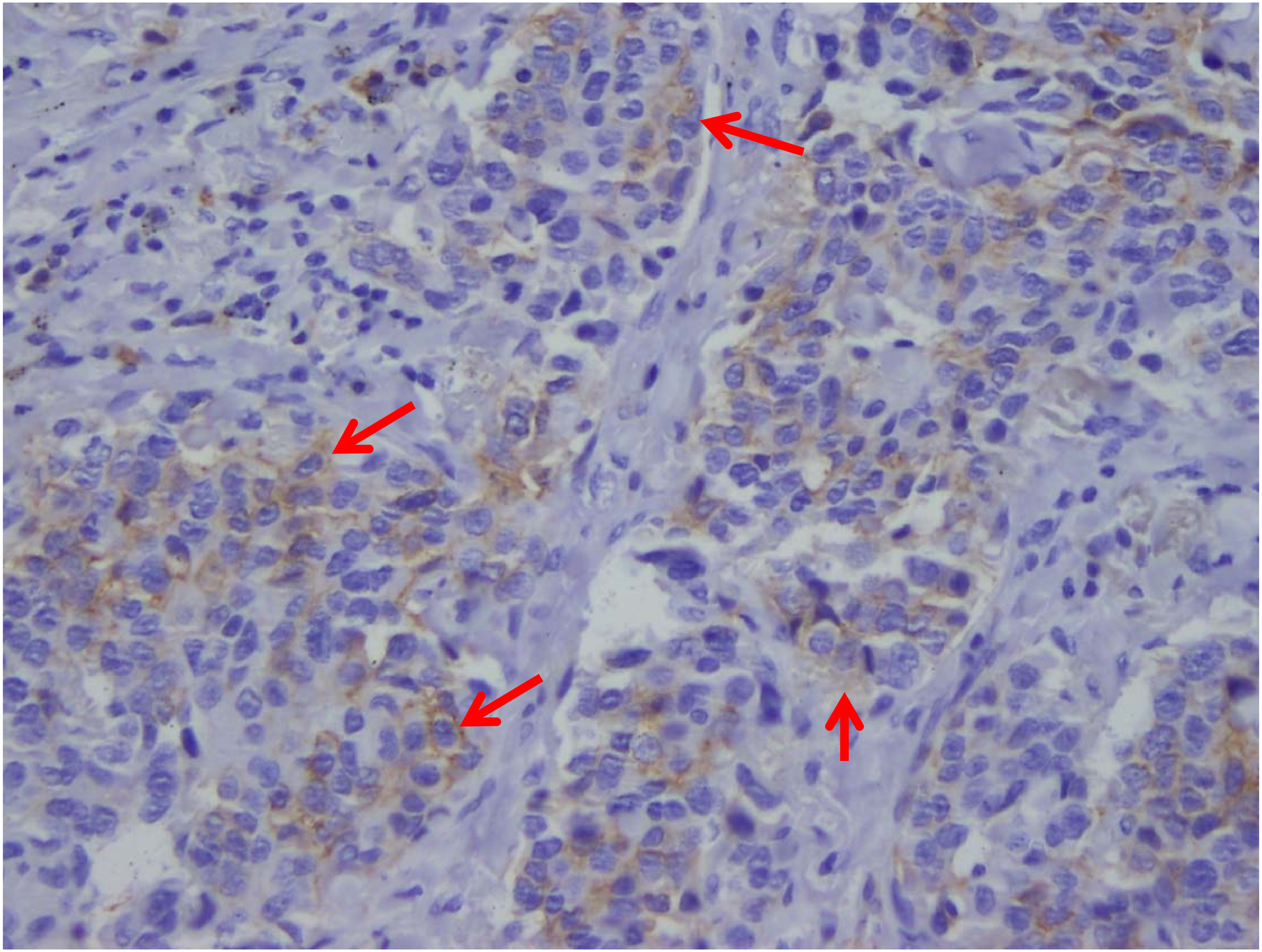

Lung adenocarcinoma with neuroendocrine differentiation (NED). SSTR2A IHC stain positivity in the tumor cells due to NED. Some tumor cells indicated with red arrows).
