# Supplementary material for: 68Ga-DOTATATE PET/CT imaging of indeterminate pulmonary nodules and lung cancer
Source: PLoS One. 2017 Feb 9;12(2):e0171301. doi: 10.1371/journal.pone.0171301 (PMC5300187; doi:10.1371/journal.pone.0171301)
Supplement: S1 Table — (PDF) [file pone.0171301.s002.pdf]

^{68}Ga-DOTATATE PET/CT Imaging of Indeterminate Pulmonary Nodules and Lung Cancer

Ronald Walker,[1,2,3] Stephen Deppen,[4] Gary Smith,[1,2] Chanjuan Shi,[5] Jonathan Lehman,[6] Jeff Clanton,[2] Brandon Moore,[1] Rena Burns,[3] Eric L Grogan,[4,7] Pierre P Massion[3,8,9*]

Affiliations:
[1]Medical Imaging Service, Tennessee Valley VA Healthcare System, Nashville, TN, United States of America
[2]Department of Radiology and Radiological Sciences, Vanderbilt University Medical Center, Nashville, TN, United States of America
[3]Vanderbilt-Ingram Cancer Center, Nashville, TN, United States of America
[4]Department of Surgery, Vanderbilt University Medical Center, Nashville, TN, United States of America
[5]Department of Pathology, Microbiology and Immunology, Vanderbilt University Medical Center, Nashville, TN, United States of America
[6]Department of Medicine, Division of Hematology/Oncology, Vanderbilt University Medical Center, Nashville, TN, United States of America
[7]Department of Surgery, Tennessee Valley Healthcare System, Nashville, TN, United States of America
[8]Department of Medicine, Division of Pulmonary, Allergy and Critical Care Medicine, Vanderbilt University Medical Center, Nashville, TN, United States of America
[9]Pulmonary Critical Care Section, Medical Service, Tennessee Valley Healthcare System, Nashville, TN, United States of America

Running title: ^{68}Ga-DOTATATE - Lung Nodules & Cancer

*Corresponding author
Email: pierre.massion@vanderbilt.edu

Key words: ^{68}Ga-DOTATATE; lung cancer; lung nodule; somatostatin receptor

Word count: 5842

Subj ID	Lesion diameter (cm)	^{18}F-FDG SUVMAX	^{68}Ga-DOTATATE SUVMAX	Age (y)	Fasting blood sugar mg/dL	Diagnosis	Cancer	Pet pos	dota pos	pet right	dota right	Gender F = 1 M = 0	Ethnicity Caucasian = 1	Details
2	1.6	1.0	0.4	60	267	Benign	0	0	0	1	1	0	1	biopsy - fibrosis and necrosis, neg. for malignant cells
183	1.0	0.5	0.5	49	92	Benign	0	0	0	1	1	0	1	Benign granuloma via follow-up imaging results.
25	1.5	4.5	0.6	79	104	Metastatic	1	1	0	1	0	0	1	RML-malignant cells present, consistent with metastatic urothelial carcinoma (patient had urothelial carcinoma 10 y ago)
218	1.5	0.7	0.8	> 80	107	Benign	0	0	0	1	1	0	1	Benign granuloma, but had microscopic focus of bronchus-associated lymphoid tissue (BALT) lymphoma
109	3.9	1.2	0.8	40	97	Benign	0	0	0	1	1	0	1	endobronchial biopsy -chronic inflammation, negative for tumor or granuloma, broncholith noted in bronchoscopy report
93	2.0	1.3	0.8	68	109	Benign	0	0	0	1	1	0	1	rare fungal yeasts, consistent with histoplasmosis, negative for malignant cells
182	1.0	1.4	0.8	46	104	Benign	0	0	0	1	1	0	1	nodules stable by 2 y CT imaging. No cytology obtained
138	2.2	5.2	0.8	50	112	ADC	1	1	0	1	0	0	1	LUL-moderately to poorly differentiated adenocarcinoma
3	1.5	1.3	0.9	46	124	Benign	0	0	0	1	1	0	1	Pulm nodules stable by 2 y follow-up CT imaging, no cytology reports
120	2.0	5.8	0.9	53	125	Benign	0	1	0	0	1	0	1	Two nodules - this one RUL - negative for malignant cells via CT biopsy and on more than 2 y CT follow-up
120	3.0	0.9	1.2	53	125	Benign	0	1	0	0	1	0	1	Two nodules - this one RLL - negative for malignant cells via CT biopsy and on more than 2 y CT follow-up
179	1.1	0.7	1.0	63	116	Benign	0	0	0	1	1	0	1	patient declined biopsy and elected CT follow-up. Nodule was stable on 2 year follow-up by CT.
70	1.2	1.9	1.0	46	103	Benign	0	0	0	1	1	0	1	patient declined biopsy and elected CT follow-up. Nodule was stable on 2 year follow-up by CT.
192	4.4	2.3	1.0	62	108	ADC	1	0	0	0	0	0	1	RUL well-differentiated adenocarcinoma with mucinous features
129	1.4	0.8	1.2	64	104	Benign	0	0	0	1	1	1	1	RUL wedge resection - 1.5 cm dense fibrotic scar tissue - dense fibrosis, negative for malignancy, via final pathology report
70	2.4	0.8	1.2	46	103	Benign	0	0	0	1	1	0	1	patient declined biopsy and elected CT follow-up. Nodule was stable on 2 year follow-up by CT.
95	4.1	3.5	1.2	57	97	Benign	0	1	0	0	1	0	1	CT-guided FNA and core biopsy megative for malignant cells, nodule unchanged on 2 year CT follow-up
186	1.8	5.1	1.3	70	98	ADC	1	1	0	1	0	0	1	NSCLC, favor adenocarcinoma
76	1.0	2.2	1.4	72	89	Benign	0	0	0	1	1	0	1	bronchoscopic biopsy negative for malignant cells
146	6.9	15.0	1.5	63	101	ADC	1	1	1	1	1	0	1	right middle lobe adenocarcinoma by pathologic diagnosis
137	5.5	6.5	1.6	65	130	ADC	1	1	1	1	1	0	1	right lower lobe - moderately differentiated adenocarcinoma by pathologic diagnosis
162	3.2	5.6	1.8	67	241	SCC	1	1	1	1	1	0	1	squamous cell carcinoma by tissue diagnosis
68	7.0	6.1	1.9	60	88	Benign	0	1	1	0	0	0	1	benign fibrosis by tissue diagnosis
153	3.8	5.1	2.1	73	102	ADC	1	1	1	1	1	0	1	poorly differentiated adenocarcinoma via core biopsy
191	7.0	11.2	2.1	62	88	SCC	1	1	1	1	1	0	1	squamous cell carcinoma by tissue diagnosis, right lower lobe
40	2.3	5.0	2.5	78	136	SCC	1	1	1	1	1	0	1	CT-guided fine needle aspiration and 4 core biopsies - poorly differentiated NSCLC - probably squamous cell carcinoma
204	3.1	15.3	2.5	65	141	SCC	1	1	1	1	1	0	1	NSCOC - poorly differentiated squamous cell carcinoma
164	1.5	9.7	2.6	64	110	ADC	1	1	1	1	1	0	1	adenocarcinoma of the right upper lobe
100	2.1	13.9	3.2	65	71	SCC	1	1	1	1	1	0	1	squamous cell carcinoma of the right upper lobe
101	3.1	3.7	3.4	59	93	SCC	1	1	1	1	1	0	1	squamous cell carcinoma of the left lower lobe, moderately differentiated
85	6.0	13.6	3.8	72	112	SCC	1	1	1	1	1	0	1	squamous cell carcinoma of the right upper lobe
						total	15	18	12	26	26	1	31	

ADC = adenocarcinoma of the lung. SCC = squamous cell lung cancer. Metastatic = a metastatic lung nodule from urinary bladder cancer.
